# Tracking Systems Used to Monitor the Performance and Activity Profile in Elite Team Sports

**DOI:** 10.3390/s21248251

**Published:** 2021-12-10

**Authors:** Jose Luis Felipe, Jorge Garcia-Unanue, Leonor Gallardo, Javier Sanchez-Sanchez

**Affiliations:** 1 School of Sport Sciences, European University of Madrid, 28670 Madrid, Spain; javier.sanchez2@universidadeuropea.es; 2 Digital Transformation and Performance Analysis Department, UD Las Palmas, 35019 Las Palmas de Gran Canaria, Spain; 3 IGOID Research Group, Department of Physical Activity and Sport Sciences, University of Castilla-La Mancha, 45071 Toledo, Spain; Jorge.GarciaUnanue@uclm.es (J.G.-U.); Leonor.Gallardo@uclm.es (L.G.)

Technology has become established in elite sport in recent years and is used on a regular basis, especially in team sports. Thereby, monitoring movement patterns reveals important results regarding sport performance. In the area of semi-professional or elite football, the best system to quantify these results and the kinematic profile of the players are Global Positioning Systems (GPS) [1]. These devices have been defined as a valid tool for evaluating the external load in intermittent sports, with capacity to record real-time data about time, speed, distance, position, altitude, and direction [2], which makes them very common in the analysis of team sports [3,4].

Furthermore, the technology of a multiple-camera match analysis system, implemented in almost all European professional leagues and international competitions, has been demonstrated to be as reliable as GPS; it can obtain results in quantification with less than 5% of error, and has been proven valid for investigation [5]. Thus, these types of systems have been used for physical and tactical performance studies in different elite competitions [6,7].

Whereas the processes underlying tactics in elite football have improved over the years, scientific approaches have not evolved with the same speed. A potential solution to this problem can be found in the big data technologies [8]. The definition of big data is usually made through the so-called three Vs: Volume, Variety, and Velocity [9]. Big data aims to provide a standardized way for researchers to access complex processing algorithms and to make it possible for non-expert users to apply cutting-edge analysis technologies to their data [10].

The progress of computing has made it possible to obtain position data about the movements of players [11]. Positioning tracking systems establish the player and ball position in the x coordinates (parallel to lateral lines) and y coordinates at 25 frames per second, equivalent to approximately 135,000 positions per subject and game, and a total of about 3,100,000 positions between all players and the ball. The compilation of all this data and the subsequent analysis will facilitate the simplification of theory and practice in sports [12]. In this sense, while physical parameters such as distance covered or speed have been already analyzed in recent years, there is little scientific evidence evaluating tactical parameters in team sports.

The use of these data together in predictive models is one of the more pressing topics for research in Sport Sciences [8]. Specifically, previous research has included variables of physical performance (maximum speed (V_max_) and medium speed (V_medium_) in 15 min intervals; distances covered at different speeds; medium distance covered, maximum speed in sprints; peak acceleration; and the number of accelerations of players in different ranges) and technical performance (number of passes, ball control, tackles, headed shots, shots on goal, corners, and free kicks by areas: defensive area (third of the field closest to the defensive zone), central area (central third of the field of game), and offensive area (third of the field of play closest to the rival goal); number of short distance passes (<10 m), average (>10 m), and minimum distance (<2 m); number of shots on goal; % ball possession; and tackles and interceptions at 15 min intervals).

To determine the tactical behavior of players or teams, a series of variables are required to demonstrate the behavioral dynamics of these agents. The centroids, depth and width, and stretch rates of the team seem to provide a solid basis for analyzing the collective behavior in attack and defense team dynamics [7]. Within these concepts, the team centroid is defined as the middle position of all the players in a team; it presents low variability when trying to measure the coordination between players in a soccer match. However, the variation between each player and their specific position (individual centroid) has been identified as a potential variable to determine the dynamic behavior of the player with greater precision [13]. From a dynamic system perspective, the high variability in the distance between teams (distance between the centroid of two teams) is expected to reflect disturbances in the balance between team behavior, which precede critical events of the game such as game goal occasions [7].

Variables can be obtained through the multiple-camera match analysis system and the other technologies mentioned above, and they allow us to differentiate physical, tactical, and technical load indicators, and they can be combined with possible new calculation variables [12].

The applicability of the information obtained by this technology can be oriented in different areas of execution:

- **Injury prevention:** Physical load control of the players is one of the major objectives that physical training departments of teams or athletes aim for. Knowing the internal and external diary load of the athlete allows training objectives to be readjusted and physical performance to be optimized. The data obtained thought the previously commented technology can be synchronized with heart rate monitoring, which facilitates the knowledge of internal load generated by training stimulus, as well as a mechanical load which the athlete is subjected to.
- **The orientation of training tasks:** The information provided by this technology allows for the identification of critical competition sceneries, in other words, the highest demands that athletes are subjected to when facing a match and/or sports competition in which their objective is to achieve the best result. The identification of these physical demands allows us to orientate training tasks as well as their planning with their adaptive process consecution; this enables the athletes to cope with these competing demands when subjected to them.
- **Technical–tactical development:** The provision of the positional data of the athletes enables one to identify their limitations and strengths regarding tactical behavior. In team sports, the interaction between the components of the same team, as well as the presence of the opposition, is essential to obtain results. For this reason, the information provided by these technological systems allows the establishment of interaction networks with the aim of knowing the tactical behavior of the team, in order to enhance the identified weaknesses and guide training tasks based on quantitative information.

In recent years, technological developments in team sports have changed the daily work in the area of sport sciences. Information provided by the tracking system reduces uncertainty in the decision-making process. In this sense, tracking systems enable the implementation of the PDCA cycle (Plan, Do, Check, and Act) to optimize the performance and prevent injuries of players. Following the principles of this model, the tracking system should allow one to identify and analyze the main problems related to the external load, locomotor demands and technical–tactical performance of any session or match (Plan), to develop and implement a holistic solution to the identified situation with the rest of the staff members (Do), to evaluate the results and the achieved goals according to the performance and injuries of the players (Check), and to standardize the strategies to help with decision making regarding players’ training programs. In conclusion, tracking systems can be considered a valid and reliable tool to implement a problem-solving model in the context of the elite team sports.

## Data Availability

Not applicable.
